# Peripheral Blood Mitochondrial DNA Copy Number Is Associated with Prostate Cancer Risk and Tumor Burden

**DOI:** 10.1371/journal.pone.0109470

**Published:** 2014-10-03

**Authors:** Weimin Zhou, Min Zhu, Ming Gui, Lihua Huang, Zhi Long, Li Wang, Hui Chen, Yinghao Yin, Xianzhen Jiang, Yingbo Dai, Yuxin Tang, Leye He, Kuangbiao Zhong

**Affiliations:** 1 Department of Urology, The Third Xiangya Hospital, Central South University, Changsha, China; 2 Department of Abdominal Surgery, Jiangxi Cancer Hospital, Nanchang, China; 3 Molecular Biology Research Center, Xiangya School of Medicine, Central South University, Changsha, China; 4 Department of Nephrology, The Third Xiangya Hospital, Central South University, Changsha, China; 5 Center for Medical Experiments, The Third Xiangya Hospital, Central South University, Changsha, China; 6 Department of Epidemiology and Statistics, Public Health School, Central South University, Changsha, China; Northern Institute for Cancer Research, United Kingdom

## Abstract

Alterations of mitochondrial DNA (mtDNA) have been associated with the risk of a number of human cancers; however, the relationship between mtDNA copy number in peripheral blood leukocytes (PBLs) and the risk of prostate cancer (PCa) has not been investigated. In a case-control study of 196 PCa patients and 196 age-paired healthy controls in a Chinese Han population, the association between mtDNA copy number in PBLs and PCa risk was evaluated. The relative mtDNA copy number was measured using quantitative real-time PCR; samples from three cases and two controls could not be assayed, leaving 193 cases and 194 controls for analysis. PCa patients had significantly higher mtDNA copy numbers than controls (medians 0.91 and 0.82, respectively; *P*<0.001). Dichotomized at the median value of mtDNA copy number in the controls, high mtDNA copy number was significantly associated with an increased risk of PCa (adjusted odds ratio  = 1.85, 95% confidence interval: 1.21–2.83). A significant dose-response relationship was observed between mtDNA copy number and risk of PCa in quartile analysis (*P*_trend_ = 0.011). Clinicopathological analysis showed that high mtDNA copy numbers in PCa patients were significantly associated with high Gleason score and advanced tumor stage, but not serum prostate-specific antigen level (*P* = 0.002, 0.012 and 0.544, respectively). These findings of the present study indicate that increased mtDNA copy number in PBLs is significantly associated with an increased risk of PCa and may be a reflection of tumor burden.

## Introduction

Human mitochondrial DNA (mtDNA) is a 16,569 bp chromosome that is double-stranded and circular in nature. It is maternally inherited and encodes for 13 core polypeptide subunits that compose the respiratory chain complexes, two rRNAs, and a set of 22 tRNAs, which are required for mitochondrial protein synthesis [1]. Compared with nuclear DNA, mtDNA lacks both introns and protective histones and also has diminished DNA repair capacity. These features make it particularly susceptible to reactive oxygen species (ROS) and other types of damage that could lead to sequence mutations or copy number alterations [2], [3]. Such alterations of mtDNA could subsequently affect the expression of mitochondrial genes as well as a wide range of mitochondrial functions such as energy production, signal transduction, cell cycle regulation, cellular differentiation, apoptosis, and growth [4]. Thus, abnormal changes in mtDNA could potentially result in deficiencies in oxidative phosphorylation, enhancements to the production of ROS during aerobic metabolism, or even result in a malignant state.

Prostate cancer (PCa) is the second most frequently diagnosed cancer and the sixth leading cause of cancer-related death in men, with an estimated 914,000 new cases and 258,000 deaths occurring per year globally [5]. Historically the incidence of PCa has been significantly lower in Asian than Caucasian males; however, in China, as lifestyles are becoming more westernized, the incidence rate of PCa has increased significantly in recent years. So far, serum prostate-specific antigen (PSA) is the best available prostate-specific tumor marker. However, there is an ongoing controversial debate on the use of serum PSA testing for PCa [6]. An estimated rate of overdiagnosis as high as 50% has been reported, and the adverse side effects related to unnecessary treatments make the overall benefit of PSA mass screening unclear [7]. Thus, the identification of additional molecular markers is needed to improve the screening and diagnosis of PCa.

Multiple retrospective and prospective studies have investigated the association of constitutive mtDNA copy number in peripheral blood leucocytes (PBLs) with the risk of cancers [8]–[21]. Until now, the relationship between mtDNA copy number in PBLs and PCa risk has not been established. In the following experiments, we utilized a retrospective case-control study in Han Chinese to evaluate the association of mtDNA copy number in PBLs with the risk of PCa. We also examined whether mtDNA copy number correlated with clinicopathological characteristics of PCa patients.

## Materials and Methods

### Study population and epidemiological data

A total of 196 patients with histologically confirmed primary prostate adenocarcinoma were consecutively recruited between January 1, 2006 and September 1, 2012 at the Third Xiangya Hospital and Hunan Provincial Tumor Hospital, both of which are affiliated with the Central South University (Changsha, China). These patients represented 84% of all new cases diagnosed at both hospitals during the study period. None of the cases had received any previous PCa-related treatment, had a history of any other types of cancer, or severe medical co-morbidities including cardiac disease, active cerebral infarction, or severe infection over the last three months. All patients underwent pre-treatment evaluation, including bone scan, chest X-ray, and either a computed tomography (CT) or magnetic resonance imaging (MRI) of the abdomen and pelvis to evaluate the tumor stage. PCa stage was classified according to the seventh American Joint Committee on Cancer (AJCC) system.

As controls, 196 age-matched (±2 years) healthy subjects from Hunan province during the same time period as the case enrollment were enrolled from the Center for Health Management of the Third Xiangya Hospital. Control subjects had a PSA level of less than 4 ng/ml, had a normal digital rectal examination, and had neither a previous history of cancer nor any of the aforementioned medical comorbidities. Approximately 82% of the individuals invited to participate as control subjects agreed to enroll in the study.

All case and control participants were Han Chinese. All were interviewed by trained interviewers to collect information including age, body mass index (BMI), smoking history, dietary habits, and family history of cancer and medical history. A never smoker was defined as an individual who never smoked or who smoked <100 cigarettes during his or her lifetime. An individual who smoked >100 cigarettes was defined as an ever smoker. Daily dietary fat intake was classified into three categories according to Chinese Dietary Reference Intake levels [22]. The percentage of daily fat energy intake of the total energy <20%, 20–30% and >30% were defined as low, moderate and high daily dietary fat intake, respectively. The following morning after the interview, 10-ml fasting blood samples were collected.

This study was approved by the Research Ethics Committee of Central South University (Changsha, China). Written informed consent was obtained from all participants. The main characteristics of our study cohort are summarized in Table 1. We were unable to assay samples from three cases and two controls, leaving 193 cases and 194 controls for our analyses.

**Table 1:** Distribution of Selected Characteristics in PCa Cases and Controls.

Characteristics	Cases (n = 193)	Controls (n = 194)	*P* value
Age, years	70.3±9.0	70.1±9.2	0.861
BMI, kg/m^2^	24.05±3.80	23.22±3.67	0.029
Smoking history, No. (%)			
Never	93 (48.2)	96 (49.5)	
Ever	100 (51.8)	98 (50.5)	0.798
Daily dietary fat intake^a^, as % of total energy, No. (%)			
Low (<20%)	49 (25.4)	67 (34.5)	
Moderate (20%–30%)	84 (43.5)	89 (34.9)	
High (>30%)	60 (31.1)	38 (19.6)	0.020
Family history of PCa, No. (%)	18 (9.3)	2 (1.0)	<0.001
AJCC stage^b^, No. (%)			
II A	13 (6.8)		
II B	16 (8.4)		
III	37 (19.5)		
IV	124 (65.3)		
PSA level, No. (%)			
<10 ng/ml	24 (12.4)		
10∼20 ng/ml	18 (9.3)		
>10 ng/ml	151 (78.2)		
Gleason score^c^, No. (%)			
5–6	58 (30.7)		
7	66 (34.9)		
8–10	65 (34.4)		

Abbreviations: PCa  =  prostate cancer; BMI  =  body mass index; AJCC  =  American Joint Committee on Cancer; PSA  =  prostate-specific antigen.

a Data were defined according to Chinese Dietary Reference Intake levels [22].

b Data of AJCC stage were classified by the 7th AJCC staging system; Data were not available for 3 cases.

c Data of Gleason score were not available for 4 cases.

### mtDNA copy number assessment by quantitative real-time PCR

High-quality genomic DNA was extracted from participants' peripheral blood using the QIAamp DNA Mini Kit (Qiagen, Valencia, CA) and according to the manufacturer's protocol. The relative mtDNA copy number was measured by quantitative real-time PCR (qPCR) as previously described [23], [24]. Briefly, two pairs of primers were designed and used for the quantification of mtDNA copy number. The first primer pair was used for the amplification of the *ND1* gene in mtDNA. The primer sequences were as follows: forward primer (ND1-F), 5′-CCCTAAAACCCGCCACATCT-3′; reverse primer (ND1-R), 5′-GAGCGATGGTGAGAGCTAAGGT-3′. The second primer pair was used for the amplification of the nuclear gene human globulin (*HGB*). The primer sequences were as follows: forward primer (HGB-1), 5′-GTGCACCTGACTCCTGAGGAGA-3′; reverse primer (HGB-2), 5′-CCTTGATACCAACCTGCCCAG-3′. The PCR reaction mixture (10 µl) for the mtDNA amplification consisted of 2× SYBR Green Mastermix (CoWin Biotech, Beijing, China), 200 nmol/l ND1-F (or HGB-1) primer, 200 nmol/l ND1-R (or HGB-2) primer, and 5 ng of genomic DNA. Thermal cycling conditions were 95°C for 10 min followed by 40 cycles at 95°C for 15 s and either 60°C (for *ND1* amplification) or 56°C (for *HGB* amplification) for 1 min. Each sample was run in triplicate in a 96-well plate with an ABI PRISM 7000 Sequence Detection System (Applied Biosystems, USA).

The qPCR procedures for *ND1* and *HGB* were performed in separate 96-well plates with the same samples in the same well positions to avoid possible position effects. During each run, a negative control (water), a positive control (calibrator DNA), and a standard curve were included. The calibrator DNA was a genomic DNA sample from a healthy control subject that was used to compare the results from different independent assays. Each plate contained randomly selected samples, thus ensuring equal representation of all cases and controls. The lab personnel were blind to case or control status. We used pooled DNA as reference DNA from 30 participants that had been randomly selected from controls in this study (200 ng of genomic DNA for each sample). For the standard curve, the reference DNA sample was serially diluted with a two-fold incremental dilution to generate a five-point standard curve. This allowed between 40 and 2.5 ng of DNA in each reaction. The *R* ^2^ correlation for each standard curve was ≥0.99, with acceptable standard deviation (SD) set at 0.25 for the Ct values. Otherwise, the sample was repeated. The ratio of the mtDNA copy number to the single gene (*HGB*) copy number was determined for each sample using standard curves. This ratio was proportional to the mtDNA copy number in each sample. The ratio for each sample was then normalized to the calibrator DNA sample to standardize between different runs. Finally, in order to assess any intra-assay variation, we assayed 10 blood DNA samples from healthy control subjects at three times on the same day. We then repeated the assay with the same blood DNA samples on three different days to evaluate inter-assay variation. Average coefficients of intra-assay and inter-assay variance were 3.6% (range, 1.1%–9.1%) and 3.9% (range, 0.8%–7.3%), respectively. These results suggest both high intra-assay and inter-assay reliability.

### Statistical analyses

All statistical calculations were carried out using SPSS 16.0 software (IBM, Chicago, IL, USA). Differences in the distribution of host characteristics between the cases and the controls were evaluated by Pearson χ^2^ test for categorical variables and, Student *t* test (age and BMI) and Mann Whitney U test (mtDNA copy number) for continuous variables. Spearman rank correlation analysis was applied in studying the relationship between mtDNA copy number and clinicopathological factors of PCa. The mtDNA copy number was also analyzed as a categorical variable using median or quartile distribution in the controls. Then unconditional multivariate logistic regression, adjusted for PCa potential risk factors including age, BMI, daily dietary fat intake, smoking status and family history of PCa, was used to evaluate the association between mtDNA copy number and the risk of PCa by estimating odds ratios (OR) and 95% confidence intervals (95%CI). Test for trend was performed using the median mtDNA copy number value for each quartile. This regression was carried out using the entire case and control groups, as well as subgroups defined by different demographic and clinicopathological characteristics. All statistical tests were two-sided, and the level of statistical significance was set at *P*<0.05.

## Results

**Figure 1:**
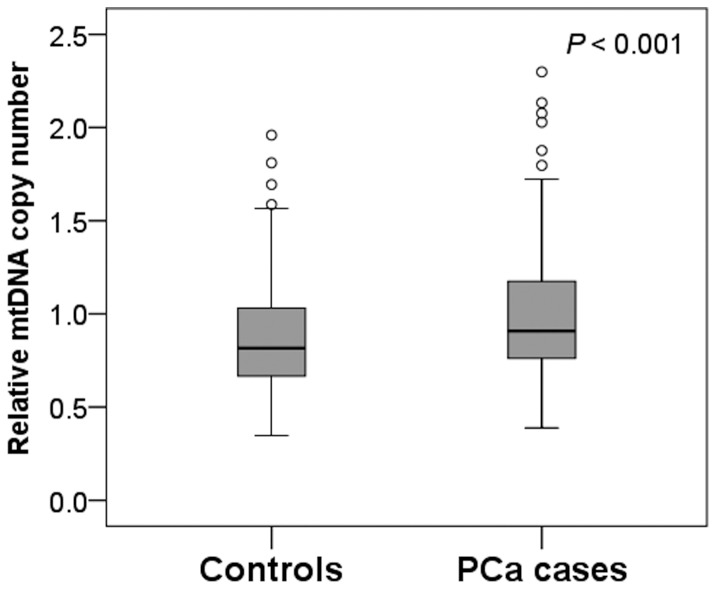
Distribution of peripheral blood mtDNA copy number in Han Chinese patients with prostate cancer and healthy controls. The box plots describe the median (solid line across the box), inter-quartile range and outliers (circles outside the ends of whiskers) for each study group. The *P*-value is from a comparison of mtDNA copy number distribution between cases and controls using the Mann Whitney U test.

A total of 196 patients with PCa and 196 healthy controls were included in this study. Samples from three cases and two controls could not be assayed, leaving 193 cases and 194 controls for analysis. The characteristics of the study population are summarized in Table 1. There were no statistically significant differences between cases and controls in age and smoking history. However, cases were more likely than controls to have higher BMI, higher daily dietary fat intake, and a family history of PCa (Table 1). Median mtDNA copy number was higher among cases than among controls (0.91 and 0.82, respectively; *P*<0.001, Figure 1).

We performed unconditional logistic regression analysis to assess the association between mtDNA copy number and PCa risk. When individuals were separated into high or low groups based on the median mtDNA copy number value in healthy controls, we observed that high mtDNA copy number was associated with an increased risk of PCa after adjusting for age, BMI, daily dietary fat intake, smoking status and family history of PCa (higher median *vs* lower, odds ratio (OR) = 1.85, 95%CI: 1.21–2.83; Table 2). Analysis of the data by the quartile distribution of mtDNA copy number in controls revealed a dose-response association between mtDNA copy number and PCa risk (highest quartile *vs* lowest: OR = 2.52, 95%CI: 1.35–4.70; *P*_trend_ = 0.011; Table 2).

**Table 2:** Risk of PCa as estimated by mtDNA copy number.

mtDNA copy number^a^	Cases (%)	Controls (%)	OR (95% CI)^b^	*P* value
By median				
≤0.82	72 (37.3)	97 (50.0)	1 (reference)	
>0.82	121 (62.7)	97 (50.0)	1.85 (1.21–2.83)	0.004
By quartile				
≤0.67	26 (13.5)	48 (24.7)	1(reference)	
0.67∼0.82	42 (21.8)	49 (25.3)	1.50 (0.78–2.88)	0.225
0.82∼1.03	57 (29.5)	49 (24.7)	2.13 (1.13–3.99)	0.019
>1.03	68 (35.2)	48 (25.3)	2.52 (1.35–4.70)	0.004
*P*_trend_				0.011

Abbreviations: PCa  =  prostate cancer; OR  =  odds ratio; mtDNA  =  mitochondrial DNA; CI  =  confidence intervals.

a mtDNA copy number was grouped based on the median/quartile value of controls.

b Analyses were performed using unconditional models adjusted for age, body mass index, daily dietary fat intake, smoking status and family history of PCa.

To determine whether the observed association between higher mtDNA copy number and PCa risk was influenced by demographic or dietary factors, we stratified the data based on age (<70 or ≥70 yr), BMI (<25 or ≥25 kg/m2), smoking status (never or ever), and daily dietary fat intake (low/moderate or high) and repeated the logistic regression. The results showed a significant relationship only among subjects who were <70 yr old, overweight (BMI ≥25 kg/m^2^), never-smokers, or those who had a low or moderate daily dietary fat intake (Table 3). However, unconditional logistic regression using the Wald test showed that the relationship between mtDNA copy number and PCa risk was not significantly affected by age (*P* = 0.127), BMI (*P* = 0.185), smoking status (*P* = 0.654) or daily dietary fat intake (*P* = 0.543).

**Table 3:** Risk of PCa as estimated by mtDNA copy number in subgroups of cases and controls with selected characteristics.

Variable	Cases, No.^a^		Controls, No.^a^		OR (95%CI)^b^	*P* value
	High	Low	High	Low		
Age, years						
<70	65	26	45	46	2.39 (1.28–4.47)	0.006
≥70	60	42	52	51	1.45 (0.83–2.53)	0.194
BMI, kg/m^2^						
<25	76	48	69	69	1.54 (0.97–2.64)	0.063
≥25	49	20	28	28	2.42 (1.14–5.16)	0.022
Smoking status						
Never	61	32	46	50	1.98 (1.09–3.61)	0.026
Ever	64	36	51	47	1.61 (0.90–2.87)	0.110
Daily dietary fat intake^c^						
Low/moderate	89	44	77	79	2.01 (1.24–3.25)	0.005
High	36	24	20	18	1.19 (0.51–2.80)	0.685

Abbreviations: PCa  =  prostate cancer; mtDNA  =  mitochondrial DNA; OR  =  odds ratio; CI  =  confidence intervals; BMI  =  body mass index.

a Cases and controls were dichotomized based on the median mtDNA copy number in controls.

b Analyses were performed using unconditional models adjusted for age, BMI, daily dietary fat intake, smoking status and family history of PCa where appropriate.

c The three categories of low, moderate and high are defined in Table 1.

To examine whether the association of mtDNA copy number with risk of PCa may reflect a role in disease progression, we explored possible correlation of mtDNA copy number with clinicopathological characteristics in PCa patients. We observed that patients with higher mtDNA copy number were more likely to have tumors in higher AJCC stages (*P* = 0.002) and to have higher Gleason scores (*P* = 0.012; Table 4). However, mtDNA copy number did not show an association with PSA level (*P* = 0.544). These results were corroborated by Spearman rank correlation analysis, which showed that mtDNA copy number correlated positively with AJCC stage (*r* = 0.260, *P*<0.001) and Gleason score (*r* = 0.216, *P* = 0.003), but it did not correlate with PSA level (*r* = 0.012, *P* = 0.872).

**Table 4:** Correlation of mtDNA copy number and clinicopathological parameters in PCa patients.

Variable	No. of patients (%)	mtDNA copy number, median (interquartile range)	*P*
PSA, ng/ml			
<10	24 (12.4)	0.88 (0.80–1.05)	
10∼20	18 (9.3)	0.89 (0.80–1.14)	
≥20	151 (78.2)	0.92 (0.74–1.20)	0.544
AJCC stage^a^			
II A + II B	29 (15.3)	0.80 (0.66–0.88)	
III	37 (19.5)	0.88 (0.72–1.01)	
IV	124 (65.3)	1.00 (0.79–1.33)	0.002
Gleason score^b^			
5–6	58 (30.7)	0.83 (0.69–1.03)	
7	66 (34.9)	0.90 (0.80–1.18)	
8–10	65 (34.4)	1.00 (0.77–1.32)	0.012

Abbreviations: mtDNA  =  mitochondrial DNA; PCa  =  prostate cancer; PSA  =  prostate-specific antigen; AJCC  =  American Joint Committee on Cancer.

a Data of AJCC stage were classified by the 7th AJCC staging system; data were not available for 3 cases.

b Data of Gleason score were not available for 4 cases.

As a further check to verify that mtDNA copy number varied with certain clinicopathological characteristics of patients with PCa, we performed unconditional logistic regression after adjusting for potential confounders, including age, PSA level, AJCC stage, and Gleason score (Table 5). Patients in AJCC stage III were more likely to have higher mtDNA copy number than those in stage II (OR = 2.93, 95%CI: 1.01–8.50), as were patients in AJCC stage IV (OR = 3.38, 95%CI: 1.33–8.57, *P*_trend_ = 0.009). Patients with a Gleason score of 7 tended to have higher mtDNA copy number than those with lower scores, but this difference was not significant (OR = 1.80, 95%CI: 0.83–3.92). A similar result was obtained for patients with Gleason scores of 8–10 (OR = 1.61, 95%CI: 0.73–3.53, *P*_trend_ = 0.063). Patients with PSA levels of 10–20 ng/ml showed similar mtDNA copy number as patients with PSA<10 ng/ml (OR = 0.66, 95%CI: 0.17–2.58), as did patients with PSA ≥20 ng/ml (OR = 0.55, 95%CI: 0.20–1.53).

**Table 5:** Distribution of mtDNA copy number among subgroups of PCa patients stratified by different levels of clinicopathological characteristics.

Variable	mtDNA high, No. (%)^a^	mtDNA low, No. (%)^a^	OR (95% CI)^b^	*P* value
PSA, ng/ml				
<10	16 (13.2)	8 (11.1)	1 (reference)	
10∼20	11 (9.1)	7 (9.7)	0.66(0.17–2.58)	0.545
≥20	94 (77.7)	57 (79.2)	0.55(0.20–1.53)	0.253
*P*_trend_				0.908
AJCC stage^c^				
II A + II B	11 (9.2)	18 (25.4)	1(reference)	
III	23 (19.3)	14 (19.7)	2.93(1.01–8.50)	0.049
IV	85 (71.4)	39 (54.9)	3.38(1.33–8.57)	0.010
*P*_trend_				0.009
Gleason score^d^				
5–6	29 (24.6)	29 (40.8)	1(reference)	
7	45 (38.1)	21 (29.6)	1.80(0.83–3.92)	0.136
8–10	44 (37.3)	21 (29.6)	1.61(0.73–3.53)	0.237
*P*_trend_				0.063

Abbreviations: mtDNA  =  mitochondrial DNA; PCa  =  prostate cancer; OR  =  odds ratio; CI  =  confidence intervals; PSA  =  prostate-specific antigen; AJCC  =  American Joint Committee on Cancer.

a mtDNA copy number was grouped based on the median mtDNA copy number in controls.

b Analyses were performed using unconditional models adjusted for age, PSA level, AJCC stage and Gleason score where appropriate.

c Data of AJCC stage were classified by the 7th AJCC staging system; data were not available for 3 cases.

d Data of Gleason score were not available for 4 cases.

## Discussion

The results of this case-control study, to our knowledge, are the first molecular epidemiological investigation of leukocyte mtDNA copy number and PCa risk. Our study suggests that high mtDNA copy number is associated with increased risk of PCa. In addition, the mtDNA copy number was positively correlated with the AJCC tumor stage and potentially with Gleason score, suggesting a role of tumor burden in determination of blood mtDNA copy number.

The biological mechanism for mtDNA copy number in PBLs and cancer risk is not completely understood. Blood cells function as transporter cells and as mediators of the immune response. Thus, blood contacts and interacts with all human tissues and can convey a range of bioactive molecules, including oxygen, nutrients and metabolites, antibodies, cytokines, and hormones [25]. Therefore, blood cell profiling represents a powerful means to explore disease pathogenesis and physiological homeostasis and, more generally, the complexity of systems biology [26]. In the past several years, multiple studies with retrospective and prospective study designs have demonstrated a strong association between constitutive mtDNA copy number in PBLs and the risk of various cancers. More specifically, increased copy number was observed in breast, pancreas, colorectum, and lung cancers, and non-Hodgkin lymphoma [8]–[13]. However, other studies have found a decrease in mtDNA copy number in breast, liver, stomach, esophageal, and renal cancers, as well as soft tissue sarcoma [14]–[20]. A U-shaped association between mtDNA copy number in PBLs and colorectal cancer risk was reported in a prospective study, with the lowest and highest quartiles both conferring a significantly increased risk of cancer when compared to the second quartile [27]. Recently, a prospective study of renal cancer revealed that high mtDNA copy number in PBLs was associated with increased future risk of renal cell carcinoma [21], which was reverse to previous two retrospective studies [18], [19]. These results may mean that mtDNA copy number correlates with cancer risk in different ways for different types of cancer. Unfortunately these results may also reflect differences in study design, experimental conditions and patient populations. Further studies should attempt to clarify these findings; in the meantime, the safest conclusion is that the precise association between mtDNA copy number and risk of cancer must be determined for each cancer individually.

Age is the most important risk factor of PCa, which is why we controlled for it in all our regression analyses. It is believed that with age, an accumulation of somatic mutations in mtDNA causes deficiencies in oxidative phosphorylation and electron transport chain, which, in turn, cause both increased production of ROS and their subsequent leakage into the cytoplasm [28]. During the aging process, elevated oxidative stress is associated with the increased abundance of mitochondria as well as the copy number and integrity of mtDNA in human cells [3], [29]. The oxidative stress and mtDNA mutations that accumulate during aging are thought to lead to higher mtDNA copy number as a compensatory mechanism [3], [30]. This process can explain only a part of the association that we observed between mtDNA copy number and risk of PCa, since we found higher mtDNA copy number to be a significant risk factor of PCa independently of age. Thus, the association observed in our study may also reflect age-independent oxidative stress. In fact, studies in humans have shown a positive association between mtDNA copy number and several markers of oxidative stress, including thiobarbituric acid-reactive substances and 8-hydroxyguanosine [31]. Increased mtDNA copy number has also been associated with lower levels of antioxidants in blood [9], [31]. These results highlight the need to explore how factors other than aging increase oxidative stress and therefore risk of PCa.

Our results, based on mtDNA copy numbers in blood cells, also support a previous tissue-based study that showed the average mtDNA content was increased in PCa tissue when compared to normal prostate tissue [32]. Two additional studies also reported that elevated mtDNA levels in the serum or plasma were present in PCa when compared to control subjects [33], [34]. Furthermore, when comparing the mtDNA copy numbers by clinicopathological characteristics, we observed that higher mtDNA copy numbers in PCa patients correlate with both higher AJCC tumor stage and Gleason score, which are the main indications that reflect the tumor burden, suggesting a possible link. This may help explain why an increase in circulating mtDNA was found to correlate with poor prognosis in PCa patients following radical prostectomy [33]. Similarly, as reported by Xia et al. [14], the content of mtDNA in whole blood in stage I breast cancer patients was significantly lower than in higher stages. Although we could not rule out the possibility of direct involvement of elevated mtDNA copy number in malignancy transformation, these lines of evidence have suggested that mtDNA may serve as a potential surrogate biomarker of tumor burden by reflecting an underlying oncogenic process, such as mtDNA mutations and oxidative stress [9].

It is worth noting that in PCa cell lines, reduction of mtDNA content leads to PCa progression, which probably through shift from androgen-dependent PCa cells to an androgen independent phenotype [35], epithelial-to-mesenchymal transition changes [36], hypermethylation of CpG islands of the putative tumor suppressor genes [37], and abnormal activation of Akt2 [38], Ras [39], ERK and JNK [36]. In fact, lower mtDNA levels in prostate tissue were found to be associated with more aggressive PCa [39]. In other types of cancers such as hepatocellular [40], gastric [41], ovarian [42] and breast cancers [36], [43], the depletion of the mitochondrial genome content was also considered as a common characteristic in cancer progression. However, in our study of PCa patients, we found a positive correlation between leukocyte mtDNA copy number and two indices of malignant progression (AJCC stage and Gleason score). These results might be caused by differing biological behavior of cancer cells and PBLs. In cancer cells, low mtDNA copy number may inhibit the respiratory chain function, resulting in a stronger tolerance to hypoxia and reducing the dependence of mitochondrial oxidative phosphorylation, thus confer tumor cells advantages of growth [39], [44], [45]. In serum or in PBLs, however, increased mtDNA copy number appears to indicate increased levels of oxidative damage that has been associated with cancer risk [9], and may reflect tumor burden that is related to malignancy degree [14], [33], rather than the direct cause of tumorigenesis.

Since repeated measures of mtDNA copy number over the treatment period in this study or previous studies were not performed, the alteration of mtDNA copy number in PBLs after treatment and during disease progression remain unclear. Therefore, future studies utilizing repeated measures may help clarify the temporal relationship between mtDNA copy number and PCa development.

Our study has several limitations that should be taken into account when applying the results. As with any retrospective case-control biomarker study, our study does not allow us to determine whether the higher mtDNA copy numbers found in PCa patients are the cause or result of cancer onset and progression. Moreover the relatively small sample size in this study limited our statistical capacity to detect interactions between mtDNA copy number and other major risk factors, such as PSA levels. Lack of statistical power may also have led to our uncertain results about the association between mtDNA copy number and Gleason score. Further prospective studies would allow confirmation of these initial findings with the use of a larger sample size. Also, our study was restricted to Han Chinese; the generalizability to other ethnic groups needs further evaluation. Finally, although we collected blood samples of newly diagnosed PCa cases prior to the start of any treatment, which should further reduce any possible impact of treatment on mtDNA copy number, of particular importance is whether treatment of PCa during progression to androgen resistance stage leads to a change of mtDNA copy number.

In conclusion, our data are the first to show that an increase in mtDNA copy number in PBLs is associated with a high PCa risk and, also, high tumor burden in PCa patients. Quantification of mtDNA copy number in PBLs could be helpful to diagnosis of PCa and assessment of tumor burden, and their potential value should be further evaluated in larger, prospective, multicenter studies.
